# Supplementary material for: Resolving the full spectrum of human genome variation using Linked-Reads
Source: Genome Res. 2019 Apr;29(4):635–45. doi: 10.1101/gr.234443.118 (PMC6442396; doi:10.1101/gr.234443.118)
Supplement: Supplemental Material [file supp_gr.234443.118_Supplemental_longranger-2.2.zip › longranger-2.2.tar.bz2!/lr2.2-public/lib/go/src/code.google.com/vitess/docs/contributing/github-workflow.html]

Vitess / GitHub Workflow


Toggle navigation

- Overview
- Guides
- Reference
- Blog
- GitHub

- #### Overview

  - What is Vitess
  - Scaling MySQL with Vitess
  - Key Concepts
- #### Getting Started

  - Run Vitess on Kubernetes
    - Custom Docker Build
  - Run Vitess Locally
- #### User Guide

  - Introduction- Client Libraries- Backing Up Data- Reparenting
        - Schema Management
        - VSchema Guide
        - Schema Swap (Tutorial)
        - Sharding
          - Horizontal Sharding (Tutorial, automated)
          - Horizontal Sharding (Tutorial, manual)
          - Sharding in Kubernetes (Tutorial, automated)
          - Sharding in Kubernetes (Tutorial, manual)
        - Vitess Sequences
        - MySQL Server Protocol
        - Vitess and Replication
        - Update Stream
        - Row Based Replication
        - Topology Service
        - Transport Security Model
        - Launching
          - Scalability Philosophy
          - Production Planning
          - Server Configuration
          - 2PC Guide
          - Troubleshooting
        - Upgrading
- #### Reference Guides

  - Vitess API- vtctl Commands
- #### Other Resources

  - Presentations- Blog- Roadmap
- #### Contributing

  - Contributing to Vitess- GitHub Workflow- Code Reviews
- GitHub

# GitHub Workflow

- #### Overview

  - What is Vitess
  - Scaling MySQL with Vitess
  - Key Concepts
- #### Getting Started

  - Run Vitess on Kubernetes
    - Custom Docker Build
  - Run Vitess Locally
- #### User Guide

  - Introduction- Client Libraries- Backing Up Data- Reparenting
        - Schema Management
        - VSchema Guide
        - Schema Swap (Tutorial)
        - Sharding
          - Horizontal Sharding (Tutorial, automated)
          - Horizontal Sharding (Tutorial, manual)
          - Sharding in Kubernetes (Tutorial, automated)
          - Sharding in Kubernetes (Tutorial, manual)
        - Vitess Sequences
        - MySQL Server Protocol
        - Vitess and Replication
        - Update Stream
        - Row Based Replication
        - Topology Service
        - Transport Security Model
        - Launching
          - Scalability Philosophy
          - Production Planning
          - Server Configuration
          - 2PC Guide
          - Troubleshooting
        - Upgrading
- #### Reference Guides

  - Vitess API- vtctl Commands
- #### Other Resources

  - Presentations- Blog- Roadmap
- #### Contributing

  - Contributing to Vitess- GitHub Workflow- Code Reviews

# GitHub Workflow

If you are new to Git and GitHub, we recommend to read this page. Otherwise, you may skip it.

Our GitHub workflow is a so called triangular workflow:

*Image Source:* https://github.com/blog/2042-git-2-5-including-multiple-worktrees-and-triangular-workflows

The Vitess code is hosted on GitHub (https://github.com/youtube/vitess).
This repository is called *upstream*.
You develop and commit your changes in a clone of our upstream repository (shown as *local* in the image above).
Then you push your changes to your forked repository (*origin*) and send us a pull request.
Eventually, we will merge your pull request back into the *upstream* repository.

## Remotes

Since you should have cloned the repository from your fork, the `origin` remote
should look like this:

```
$ git remote -v
origin  git@github.com:<yourname>/vitess.git (fetch)
origin  git@github.com:<yourname>/vitess.git (push)
```

To help you keep your fork in sync with the main repo, add an `upstream` remote:

```
$ git remote add upstream git@github.com:youtube/vitess.git
$ git remote -v
origin  git@github.com:<yourname>/vitess.git (fetch)
origin  git@github.com:<yourname>/vitess.git (push)
upstream        git@github.com:youtube/vitess.git (fetch)
upstream        git@github.com:youtube/vitess.git (push)
```

Now to sync your local `master` branch, do this:

```
$ git checkout master
(master) $ git pull upstream master
```

Note: In the example output above we prefixed the prompt with `(master)` to
stress the fact that the command must be run from the branch `master`.

You can omit the `upstream master` from the `git pull` command when you let your
`master` branch always track the main `youtube/vitess` repository. To achieve
this, run this command once:

```
(master) $ git branch --set-upstream-to=upstream/master
```

Now the following command syncs your local `master` branch as well:

```
(master) $ git pull
```

## Topic Branches

Before you start working on changes, create a topic branch:

```
$ git checkout master
(master) $ git pull
(master) $ git checkout -b new-feature
(new-feature) $ # You are now in the new-feature branch.
```

Try to commit small pieces along the way as you finish them, with an explanation
of the changes in the commit message.
Please see the Code Review page for more guidance.

As you work in a package, you can run just
the unit tests for that package by running `go test` from within that package.

When you're ready to test the whole system, run the full test suite with `make
test` from the root of the Git tree.
If you haven't installed all dependencies for `make test`, you can rely on the Travis CI test results as well.
These results will be linked on your pull request.

## Sending Pull Requests

Push your branch to the repository (and set it to track with `-u`):

```
(new-feature) $ git push -u origin new-feature
```

You can omit `origin` and `-u new-feature` parameters from the `git push`
command with the following two Git configuration changes:

```
$ git config remote.pushdefault origin
$ git config push.default current
```

The first setting saves you from typing `origin` every time. And with the second
setting, Git assumes that the remote branch on the GitHub side will have the
same name as your local branch.

After this change, you can run `git push` without arguments:

```
(new-feature) $ git push
```

Then go to the repository page and it
should prompt you to create a Pull Request from a branch you recently pushed.
You can also choose a branch manually.

## Addressing Changes

If you need to make changes in response to the reviewer's comments, just make
another commit on your branch and then push it again:

```
$ git checkout new-feature
(new-feature) $ git commit
(new-feature) $ git push
```

That is because a pull request always mirrors all commits from your topic branch which are not in the master branch.

Once your pull request is merged:

- close the GitHub issue (if it wasn't automatically closed)
- delete your local topic branch (`git branch -d new-feature`)

Contact: vitess@googlegroups.com  **·**  
Announcements  **·**  
© 2017 Vitess powered by Google Inc
